# Polydatin suppresses nucleus pulposus cell senescence, promotes matrix homeostasis and attenuates intervertebral disc degeneration in rats

**DOI:** 10.1111/jcmm.13848

**Published:** 2018-08-30

**Authors:** Jianle Wang, Chongan Huang, Zongze Lin, Xiangxiang Pan, Jiaoxiang Chen, Gang Zheng, Naifeng Tian, Yingzhao Yan, Zengjie Zhang, Jianing Hu, Pu Cheng, Xiangyang Wang, Xiaolei Zhang

**Affiliations:** ^1^ Department of Orthopaedics The Second Affiliated Hospital and Yuying Children's Hospital of Wenzhou Medical University Wenzhou China; ^2^ Zhejiang Provincial Key Laboratory of Orthopaedics Wenzhou China; ^3^ The Second School of Medicine Wenzhou Medical University Wenzhou China; ^4^ Department of paediatrics The Third Affiliated Hospital and Ruian People's Hospital of Wenzhou Medical University Wenzhou China; ^5^ Chinese Orthopaedic Regenerative Medicine Society Hangzhou China

**Keywords:** extracellular matrix, intervertebral disc degeneration, mitochondria, Nrf2, polydatin, senescence

## Abstract

Intervertebral disc degeneration (IVDD) is one of the major causes of low back pain. Polydatin (PD) has been shown to exert multiple pharmacological effects on different diseases; here, we test the therapeutic potential of PD for IVDD. In in‐vitro experiments, we confirmed PD is nontoxic to nucleus pulposus cells (NPCs) under the concentration of 400 μmol/L. Furthermore, PD was able to decrease the level of senescence in TNF‐α‐treated NPCs, as indicated by β‐gal staining as well as senescence markers p53 and p16 expression. In the aspect of extracellular matrix (ECM), PD not only reduced metalloproteinase 3 (MMP‐3), metalloproteinase 13 (MMP‐13) and a disintegrin‐like and metalloproteinase thrombospondin type 1 motif 4 (ADAMTS‐4) expression, but also increased aggrecan and collagen II levels. Mitochondrion is closely related to cellular senescence and ECM homeostasis; mechanistically, we found PD may rescue TNF‐α‐induced mitochondrial dysfunction, and it may also promote Nrf2 expression and activity. Silencing Nrf2 partly abolished the protective effects of PD on mitochondrial homeostasis, senescence and ECM homeostasis in TNF‐α‐treated NPCs. Correspondingly, PD ameliorated IVDD in rat model by promoting Nrf2 activity, preserving ECM and inhibiting senescence in nucleus pulposus cells. To sum up, our study suggests that PD exerts protective effects in NPCs against IVDD and reveals the underlying mechanism of PD on Nrf2 activation in NPCs.

## 1 INTRODUCTION

Intervertebral disc degeneration (IVDD) is one of the major diseases causing low back pain, which pervasively affects up to 80% of adults during their lives.^1^, ^2^ Intervertebral disc (IVD) is composed of nucleus pulposus (NP), annulus fibrosus (AF) and cartilaginous endplate. As the most hydrated region in IVD, NP enables the IVD to absorb compressive forces depending on the extracellular matrix (ECM) secreted by nucleus pulposus cells (NPCs).^3^ Thus, NPCs are essential to block the degradation of ECM. IVDD is a multifactorial process characterized by intracellular and biochemical changes in intervertebral discs (IVD) cells which lead to structural deterioration of IVDs. Although the pathogenesis of IVDD is not fully understood, tumour necrosis factor (TNF)‐α is reported to play a crucial role in the development of IVDD.^4^

It has been confirmed that TNF‐α is plentifully expressed in IVDD,^5^ and it is involved in diverse processes such as senescence^6^ and ECM destruction.^4^ The administration of TNF‐α aggravates the NPCs senescence by promoting levels of senescence markers p53 and p16.^6^ TNF‐α also disturbs the balance between synthesis and degradation of ECM by up‐regulating MMP‐3, MMP‐13 and ADAMTS‐4, as well as down‐regulating aggrecan and collagen II.^7^ Moreover, it is widely recognized that TNF‐α induces mitochondrial dysfunction by increasing ROS production,^8^, ^9^, ^10^ which may further aggravate senescence and ECM catabolism in NPCs.^11^, ^12^ From these studies, it is obvious that maintaining mitochondrial homeostasis would help to reverse senescence and ECM degradation in NPCs against IVDD.

Nuclear factor (erythroid‐derived 2)‐like 2 (Nrf2), a redox‐sensitive transcription factor, is the main regulator of antioxidant defence system via activating a series of cytoprotective genes against stress.^13^ Kelch‐like ECH‐associated protein 1 (Keap1), a sensor for diverse stresses as well, regulates the activity of Nrf2. During normal conditions, Keap1 ubiquitinates Nrf2 resulting in the degradation of Nrf2 via proteasome pathway; when inhibited, Keap1 loses its capacity of ubiquitinating Nrf2, allowing Nrf2 to translocate into nucleus and subsequently enhancing the expression of the antioxidant protein haeme oxygenase‐1 (HO‐1). Overexpression of HO‐1 can protect cells against oxidative stress and the protective properties are diminished when HO‐1 is inhibited,^14^ suggesting that enhanced HO‐1 could scavenge ROS and attenuate mitochondrial dysfunction induced by TNF‐α. All these findings indicate that Nrf2 is a potential therapeutic target to rescue mitochondrial dysfunction.

Polydatin (PD), a resveratrol glucoside, is abundant in different daily nourishment such as red wines, grapes as well as cocoa‐containing products.^15^, ^16^ Despite the identification of diverse beneficial functions of resveratrol (RSV) (eg anti‐inflammation, antioxidant and anti‐ageing), RSV does not exert favourable pharmacological properties because of its quick clearance rate and poor oral bioavailability.^17^, ^18^ Therefore, it is essential to search novel molecules that have extensive pharmacological protective effects beyond RSV. Similar to RSV structurally, PD possesses multiple pharmacological properties of antioxidation, anti‐inflammation and antitumour.^19^ Also, the property of PD on Nrf2 activation has been proved in different diseases models but not IVDD.^20^, ^21^, ^22^

In this study, we report that PD exerts properties of antisenescence and anti‐ECM catabolism in NPCs treated with TNF‐α. Further, we discover that PD decreases the ROS production and protects NPCs from mitochondrial dysfunction induced by TNF‐α. To explore the mechanism of PD on cytoprotection in NPCs, we demonstrate that PD activates Nrf2 and up‐regulates the level of HO‐1 in a dose‐dependent manner, whereas Nrf2 knockdown partly abolishes the protective effects in NPCs treated with TNF‐α. In rat IVDD models, oral administration of PD ameliorates the development of IVDD. We believe that this study may help us further understand the mechanism of PD on IVDD and enrich the medical therapeutic strategy against IVDD.

## 2 MATERIALS AND METHODS

### 2.1 Reagents and antibodies

Polydatin (purity > 98%) was purchased from Shanghai Aladdin Biological Technology Co, Ltd (Nantong, China). Dimethylsulphoxide (DMSO), carboxymethylcellulose (CMC) and type II collagenases were acquired from Sigma‐Aldrich (St Louis, MO). Recombinant rat TNF‐α was purchased from PeproTech (Rocky Hill, New Jersey, USA). The primary antibody against MCL‐1, cleaved caspase‐3, collagen II, aggrecan, MMP‐13, MMP‐3, ADAMTS‐4, Nrf2, HO‐1, Keap1 and Alexa Fluor^®^488‐labelled goat antirabbit IgG (H+L) second antibody and Alexa Fluor^®^594 goat antimouse IgG (H+L) second antibody were purchased from Abcam (Cambridge, UK). p16 and p53 antibodies were purchased from Sigma‐Aldrich. Antibody LC3 purchased from Cell Signaling Technology (Danvers, MA) Recombinant human TNF‐α was obtained from PeproTech and goat antirabbit, and antimouse IgG‐HRP were from Bioworld (Minneapolis, Minnesota, USA). The 4′, 6‐diamidino‐2‐phenylindole (DAPI) was purchased from Beyotime (Shanghai, China). Cell culture reagents were obtained from Gibco (Grand Island, NY).

### 2.2 Extraction and culture of rat nucleus pulposus cells

The gel‐like NP tissues were collected from tails of 4 weeks old pups (Sprague‐Dawley rats, either sex). The NP tissues were digested in 0.2% type II collagenase (Sigma) for 4 hours at 37°C. After washing with PBS, the digested tissues were transferred to DMEM/F12 (Gibco, Invitrogen, Grand Island, NY) with 15% foetal bovine serum (FBS; Gibco, Invitrogen) and antibiotics (1% streptomycin/penicillin) in the incubator at 5% CO_2_ at 37°C. When confluent, the cells were passaged after trypsinizing with 0.25% Trypsin‐EDTA (Gibco, Invitrogen) and replanted into 10‐cm culture plates at the appropriate density. We used the first three‐passage cells in our experiments.

### 2.3 Experimental design

In vitro, cells were pretreated with 50 ng/mL TNF‐α, additionally combined or alone with different concentrations of PD (0, 200, 400 μmol/L) while the control group was untreated. Cells were harvested for further experiments after 24 hours of incubation.

In vivo*,* rats were divided randomly into three groups (the vehicle group, the IVDD group and the PD group). Rats in the vehicle group and the PD group were pretreated with CMC and 50 mg/kg PD (dissolved in CMC) per day by intragastric administration, respectively, before all rats underwent puncture‐induced IVDD as described in the “Animal model” part. After puncture, rats in the vehicle group and the PD group were treated with PD as before. Rats were killed after 4 weeks postsurgery, and IVD tissues were collected for imaging, histological and immunofluorescence analysis.

### 2.4 Cell viability assay

**Figure 1:**
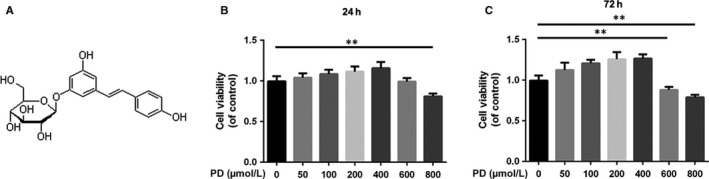
Effects of PD on the viability of NPCs. A, Chemical structure of PD. B, The cytotoxic effect of PD on NPCs was determined at various concentrations for 24 h using a CCK8 assay. C, The cytotoxic effect of PD on NPCs was determined at various concentrations for 72 h using a CCK8 assay. The values presented are the means ± SD of three independent experiments. *P < 0.05, **P < 0.01, n = 5

According to the manufacturer's protocol, cell viability was detected using the cell counting kit‐8 (CCK‐8; Dojindo Co, Kumamoto, Japan). NPCs were treated with PD and TNF‐α as described in Figure 1. After washing the cells with PBS, 100 μL of DMEM/F12 containing 10 μL of CCK‐8 solution was added into each well. Then, the plate was incubated for approximately 1 hour. The absorbance of the wells was measured using a microplate reader at 450 nm.

### 2.5 Western blotting

Nucleus pulposus cells were lysed in ice‐cold RIPA with 1 mmol/L PMSF (Phenylmethanesulphonyl fluoride, Beyotime). Protein concentrations of samples were measured by the BCA protein assay kit (Beyotime). Proteins of NPCs were separated on sodium dodecyl sulphate‐polyacrylamide gel electrophoresis (SDS‐PAGE) and were transferred to polyvinylidene difluoride membrane (Millipore, St Louis, MO, USA) followed by blocking with 5% nonfat milk. After that, the bands were probed with primary antibodies specific to aggrecan (1:1000), MMP‐3 (1:1000), MMP‐13 (1:1000), ADAMTS‐4 (1:1000), Nrf2 (1:1000), Keap1 (1:1000), HO‐1 (1:1000), p53 (1:1000), p16 (1:1000) and β‐actin (1:1000) overnight at 4°, before incubated with respective secondary antibodies. Last, the intensity of these bands was quantified using Image Lab 3.0 software (Bio‐Rad, Carlsbad, California, USA).

### 2.6 Immunofluorescence

Samples were blocked by 10% goat serum for 60 minutes at room temperature. Primary antibodies against collagen II (1:100), Nrf2 (1:200), p53(1:100) and p16 (1:100) were applied to the incubation of samples at 4° overnight. Then, the slides were incubated with Alexa Fluor^®^488‐labelled or Alexa Fluor^®^594‐conjugated second antibodies (1:400) for 1 hour and labelled with DAPI for 5 minutes. Last, slides were observed in a fluorescence microscope (Olympus Inc., Tokyo, Japan) and a confocal fluorescence microscope (Nikon, Japan). Image J software 2.1 (Bethesda, MD, USA) was used for quantification of images.

### 2.7 Cell proliferation assay

According to the manufacturer's instructions, NPCs proliferation was evaluated by the uptake of 5‐ethynyl‐2′‐deoxyuridine (EdU) into DNA, using a Click‐iT EdU microplate assay kit (Invitrogen). First, after the incubation with different test compounds as described, NPCs were labelled with EdU that was coupled to Oregon Green azide. Next, EdU incorporated into DNA was detected using HRP‐conjugated anti‐Oregon Green antibody and Amplex UltraRed. Last, samples were observed in a fluorescence microscope (Olympus Inc.).

### 2.8 Sa‐β‐gal staining

After washed with PBS twice, cells on plates were fixed with 0.2% glutaraldehyde for 10 minutes at room temperature. Then, cells were stained with X‐gal staining solution overnight at pH 6.0. Images were captured using Olympus IX71 microscope and the percentages of SA‐β‐gal‐positive cells quantified for statistical analysis.

### 2.9 Superoxide anion detection assay

The content of intracellular superoxide anion was detected using a red‐fluorescent dye, MitoSOX (Life Technologies, Carlsbad, California, USA), which stains superoxide anion in live cells and accumulates as superoxide anion‐dependent manner, at the concentration of 5 μmol/L for 45 minutes at 37°. Then, nuclei were stained with Hoechst 33 342 dye for 10 minutes at 37°. Last, samples were observed in a fluorescence microscope (Olympus Inc.) and fluorescence intensity was quantified using Image J software 2.1.

### 2.10 Mitochondrial membrane potential assay

The mitochondrial transmembrane potential was detected using a green‐fluorescent dye, MitoTracker Green (Molecular ProbesTM, Thermo Fisher Scientific Inc., Waltham, Massachusetts, USA), which stains mitochondria in live cells and accumulates as MMP‐dependent manner, at the concentration of 50 nmol/L for 30 minutes at 37°. Then, nuclei were stained with Hoechst 33 342 dye for 10 minutes at 37°. Last, samples were observed in a fluorescence microscope (Olympus Inc.) and fluorescence intensity was quantified using Image J software 2.1.

### 2.11 ATP level assay

To assess the cell ATP levels in different groups, the ATP‐GloTM Bioluminometric Cell Viability Assay (Biotium, Hayward, CA) was used according to the manufacturer's protocol. The data were collected from multiple replicate wells for each experiment.

### 2.12 Lipid peroxidation MDA assay

To assess the cell MDA levels in different groups, the Lipid Peroxidation MDA Assay Kit (Beyotime) was used according to the manufacturer's protocol. The data were collected from multiple replicate wells for each experiment with microplate reader (Bio‐Rad).

### 2.13 siRNA transfection

siRNA for rat Nrf2 gene was purchased from Santa Cruz. NPCs were seeded in six‐well plate and cultured for 50 nmol/L negative control or siRNA duplexes using Lipofectamine 2000 siRNA transfection reagent (Thermo Fisher, Waltham, Massachusetts, USA).

### 2.14 Magnetic resonance image method

The IVD signal and structural changes were detected with MRI in sagittal T2‐weighted images using a 3.0 T clinical magnet (Philips Intera Achieva 3.0MR). The parameters of T2‐weight imaging were referred to the previous study.^23^ According to the MRIs, the IVDD degree of rats was evaluated as the Pfirrmann grading system.^24^

### 2.15 Surgical procedure

The protocol for animal care and use was according to the Guide for the Care and Use of Laboratory Animals of the National Institutes of Health and was approved by the Animal Care and Use Committee of Wenzhou Medical University (ethic code: wydw2014‐0129). Rats (n = 21) were divided randomly into three groups (n = 7 per group, control group, the IVDD group and the PD group) as the above‐mentioned disposal. The PD group also was given the model operation as IVDD group. After anaesthetizing with 2% (w/v) pentobarbital (50 mg/kg), the specific level of rat tail disc (Co7/8) was located by palpation on the coccygeal vertebrae and the disc location was confirmed with a X‐ray radiograph. Needles (27G) were applied to puncture the AF through the tail skin perpendicularly, and the depth of the puncture was 4 mm according to the previous study.^11^ Needles were kept in the disc for 1 minute.

### 2.16 Alcian blue staining assay

Rats were executed with an intraperitoneal lethal dose injection of pentobarbital and tails were harvested. After fixed in formaldehyde and decalcified, the specimens were dehydrated, embedded in paraffin and cut into 5 μmol/L sections. Alcian blue staining was performed as the manufacturer's protocol. Briefly, sections were incubated with Reagent A at room temperature for 3 minutes. After removing the Reagent A, sections were incubated with Reagent B at room temperature for 30 minutes away from light. Then, after washing with double distilled water for 5 minutes, sections were stained with Reagent C at room temperature for 5 minutes away from light. Afterwards, sections were washed with double distilled water for 1 minute. Images were captured with the Olympus IX71 microscope (×40 magnification).

### 2.17 Statistical analysis

All the experiments were performed at least three times. The results were expressed as mean ± SD. Raw statistical analyses were processed using SPSS (Chicago, Illinois, USA) statistical software program 20.0. Data were analysed by one‐way analysis of variance (ANOVA) followed by the Tukey's test for comparison between control and treatment groups. Nonparametric data (Pfirrmann MRI grade scores) were analysed by the Kruskal‐Wallis H test. *P*‐value < 0.05 was considered statistically significant.

## 3 RESULTS

### 3.1 Effects of PD on the viability of rat NPCs

The chemical structure of PD is shown in Figure 1A. NPCs were treated with different concentrations of PD (0, 50, 100, 200, 400, 600, 800 μmol/L) to assess the cytotoxic effect of PD after 24 and 72 hours using Cell Counting Kit‐8 (CCK‐8). PD reduced cell viability significantly at 600 μmol/L after 24 and 72 hours (*P* < 0.01 vs untreated cells), suggesting that cell viability was not affected by ≤400 μmol/L PD at 24 and 72 hours (Figure 1B,C). Hence, 0, 200 and 400 μmol/L PD were used in further experiments.

### 3.2 Effect of PD on senescence in TNF‐α‐treated rat NPCs

**Figure 2:**
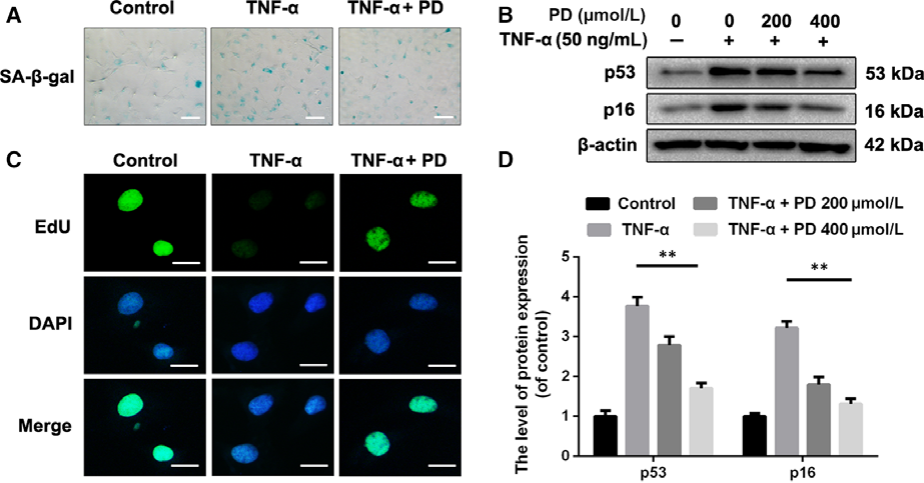
Polydatin (PD) protects NPCs from senescence induced by TNF‐α. A, Representative micrographs showed the effects of PD on senescence in NPCs treated with 10 ng/mL TNF‐α for 72 h, which was manifested by the SA‐β‐gal staining assay. Scale bar: 100 μm. B, D, The expression of p53 and p16 was detected by Western blotting. NPCs treated with or without preadministration of PD for 2 h were administrated with 50 ng/mL TNF‐α for another 72 h. C, NPCs were treated with PD or TNF‐α as described above and incubated with EdU (10 μmol/L). Then, NPCs were fixed and stained with the antibody for EdU, and nuclei were stained with DAPI. Scale bar: 10 μm. The values presented are the means ± SD of three independent experiments. *P < 0.05, **P < 0.01, n = 5

We next determined the effect of PD on the senescence in NPCs using SA‐β‐gal staining, one classical assay for detecting senescent cells. As shown in Figure 2A, PD reduced the number of SA‐β‐gal‐positive cells in TNF‐α‐treated NPCs. The levels of senescence‐related markers, p53 and p16, were detected by Western blot. Similarly, PD inhibited the TNF‐α‐induced up‐regulation of p53 and p16 (Figure 2B,C). EdU assay is widely used for detecting the capacity of proliferation, which is down‐regulated in senescent cells.^6^ We found that the administration of PD rescued the impaired capacity of proliferation in a dose‐dependent manner (Figure 2D). These data indicate that PD exerts antisenescence property in NPCs.

### 3.3 Effect of PD on anabolism and catabolism of ECM in TNF‐α‐treated rat NPCs

**Figure 3:**
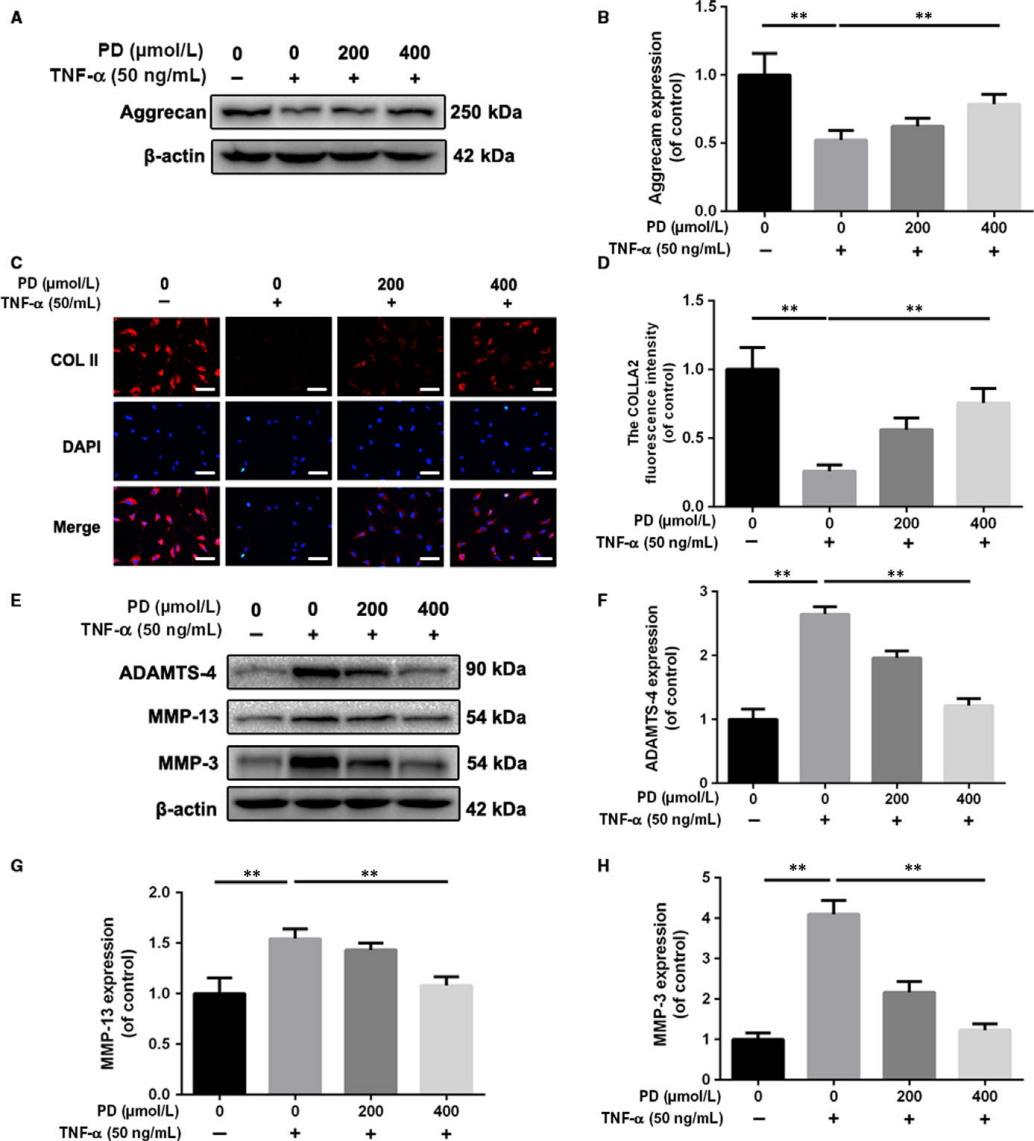
Polydatin (PD) suppresses extracellular matrix degradation induced by TNF‐α in NPCs. NPCs were pretreated with different concentrations of PD before administration with 50 ng/mL TNF‐α for another 72 h. A, B, The expressions of aggrecan were detected by Western blot. C, D, The expression of collagen II (COL II) in NPCs treated as above was detected by immunofluorescence. Scale bar: 50 μm. E‐H, The expression of MMP‐3, MMP‐13 and ADAMTS‐4 was detected by Western blot analysis. Data presented are the means ± SD of three independent experiments. *P < 0.05, **P < 0.01 vs control group; *P < 0.05, **P < 0.01, n = 5

ECM destruction, induced by excessive catabolism and inadequate anabolism, is considered as one of the characters representing degeneration in chondrocytes.^25^ To examine the protective effects of PD on anabolism of ECM, we found that TNF‐α both decreased the aggrecan level and the collagen II level, which were reversed partly with the administration of PD (Figure 3A‐D). Moreover, catabolism markers of ECM, such as ADAMTS‐4, MMP‐13 and MMP‐3, were detected by Western blot analysis. Under TNF‐α‐induced inflammatory environment, PD was able to rescue the reduction in ADAMTS‐4, MMP‐13 and MMP‐3 levels in a dose‐dependent manner (Figure 3E‐H).

### 3.4 PD prevents TNF‐α‐induced mitochondrial dysfunction in rat NPCs

**Figure 4:**
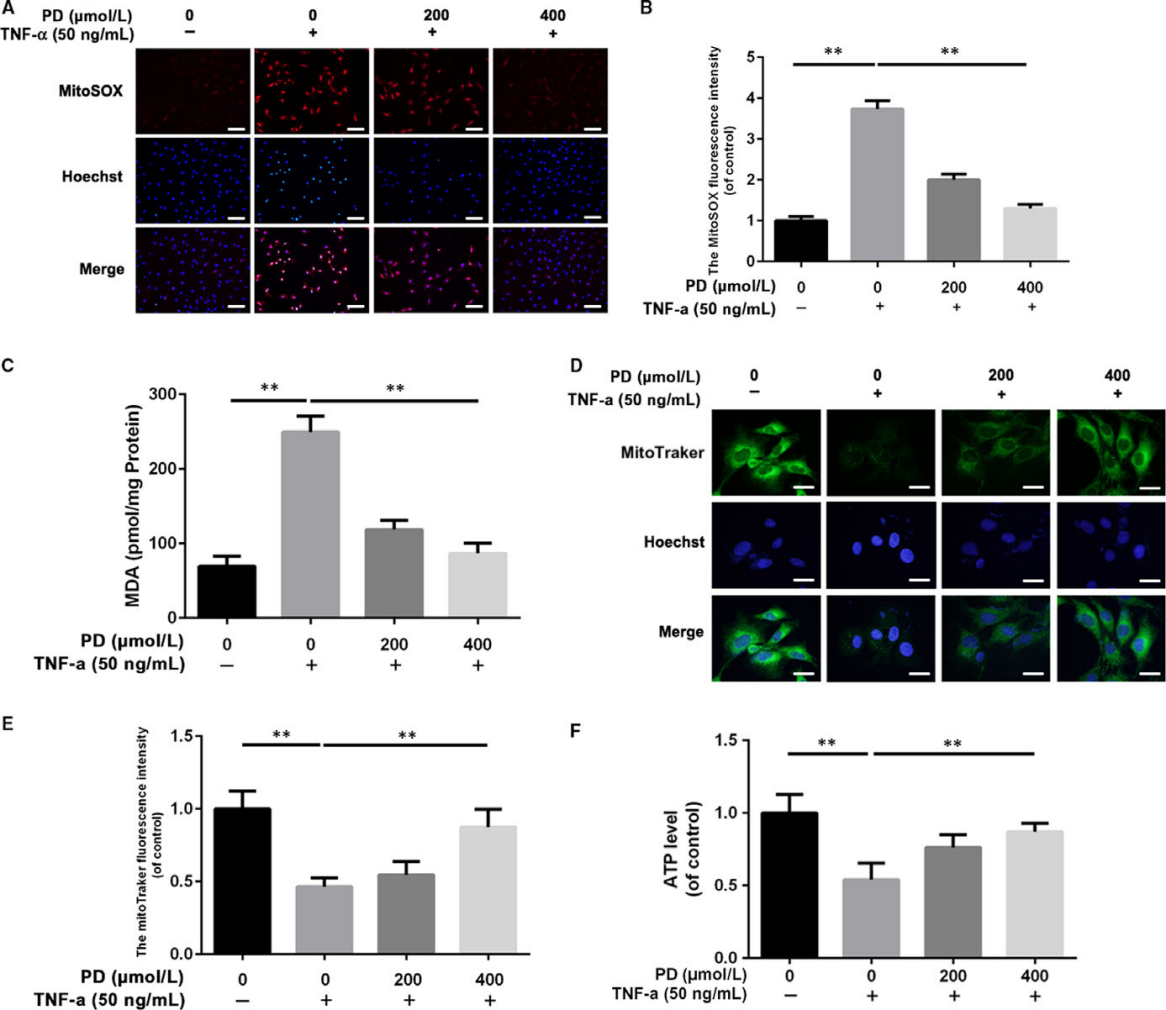
Polydatin (PD) alleviates TNF‐α‐induced mitochondrial dysfunction in NPCs. After 2 h of pretreatment with PD, TNF‐α was added into culture media at the concentration of 50 ng/mL and NPCs were incubated for another 72 h. A, B, MitoSOX assay showed that PD reversed the ROS accumulation in TNF‐α‐treated NPCs. Scale bar: 50 μm. C, Intracellular MDA level was assessed by MDA Assay Kit. D, E, The effect of PD on mitochondrial potential in NPCs treated with TNF‐α using MitoTracker Green. Scale bar: 10 μm. F, Intracellular ATP level was assessed by ATP‐Glo Bioluminometric Cell Viability Assay. The values presented are the means ± SD of three independent experiments. *P < 0.05, **P < 0.01, n = 5

TNF‐α is able to induce more ROS to aggravate mitochondrial dysfunction characterized by lower mitochondrial membrane potential and less ATP production.^10^, ^26^ MitoSOX fluorescence of NPCs was increased in the TNF‐α group compared with the control group, suggesting TNF‐α is capable of aggravating oxidative stress NPCs. After pretreatment of PD for 2 hours, the MitoSOX fluorescence decreased significantly compared with the TNF‐α group, which indicated PD could facilitate ROS scavenge in NPCs (Figure 4A,B). Also, the MDA assay showed that PD was able to alleviate the degree of oxidative stress in NPCs (Figure 4C). MitoTracker Green is one green‐fluorescent dye dependent on mitochondrial membrane potential and stains mitochondria in live cells. As shown in Figure 4D,E, 24 hours of exposure to TNF‐α led to lower MitoTracker Green fluorescence intensity in NPCs than the untreated group while 2 hours of pretreatment with PD rescued the loss of fluorescence intensity. In addition, we found that TNF‐α exposure decreased ATP level, which was reversed by PD pretreatment for 2 hours (Figure 4F). Our data show PD reverses mitochondrial dysfunction induced by TNF‐α in NPCs.

### 3.5 PD promotes Nrf2 nucleus translocation and activates the Nrf2/HO‐1 signalling pathway in NPCs

**Figure 5:**
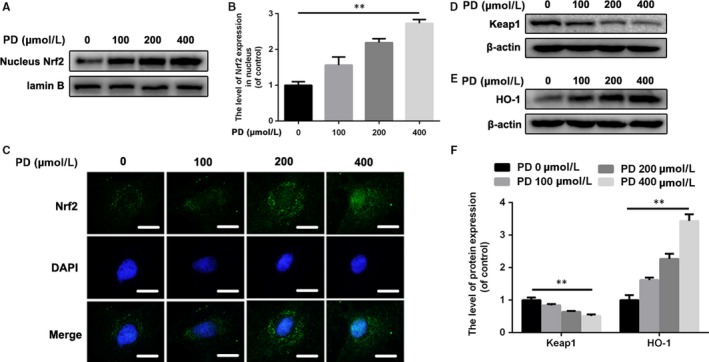
Polydatin (PD) promotes nucleus translocation of Nrf2 and activate the Nrf2/HO‐1 signalling pathway in rat NPCs. A, B, The expressions of nucleus Nrf2 in NPCs treated with different concentration of PD for 6 h. C, Representative images of Nrf2 in NPCs with increasing concentration of PD treatment for 6 h. Scale bar: 10 μm. D, The level of Keap1 was detected by Western blot. NPCs were administrated with PD for 6 h. E, The level of HO‐1 was detected by Western blot. NPCs were administrated with PD for 12 h. F, The ratios of Keap1/β‐actin and HO‐1/β‐actin were quantified. Data presented are the means ± SD of three independent experiments. *P < 0.05, **P < 0.01, n = 5

Based on the ROS scavenging property of PD, we hypothesized that PD could activate the classical Nrf2/HO‐1 antioxidative signalling pathway in NPCs. To explore the mechanism of PD on Nrf2 activation in NPCs, we detected the levels of Nrf2 in nucleus. As shown in Figure 5A,B, the levels of nucleus Nrf2 increased in NPCs treated with increasing concentration of PD for 2 hours. Also, the Nrf2 immunofluorescence showed that PD facilitated the translocation of Nrf2 into nucleus as a dose‐dependent manner (Figure 5C). To confirm the Nrf2 activation property of PD, we need observe Nrf2‐related proteins in NPCs treated with PD. We found that PD could down‐regulate the level of Keap1 in NPCs, indicating less Nrf2 was sequestered by Keap1 and more free Nrf2 could translocate into nucleus. Moreover, the level of HO‐1 was up‐regulated in NPCs treated with PD, suggesting PD is able to activate Nrf2/HO‐1 signalling pathway in NPCs (Figure 5D‐F).

### 3.6 Nrf2 mediates the beneficial effects of PD on antisenescence, anti‐ECM disorder and antimitochondrial dysfunction in NPCs treated with TNF‐α

**Figure 6:**
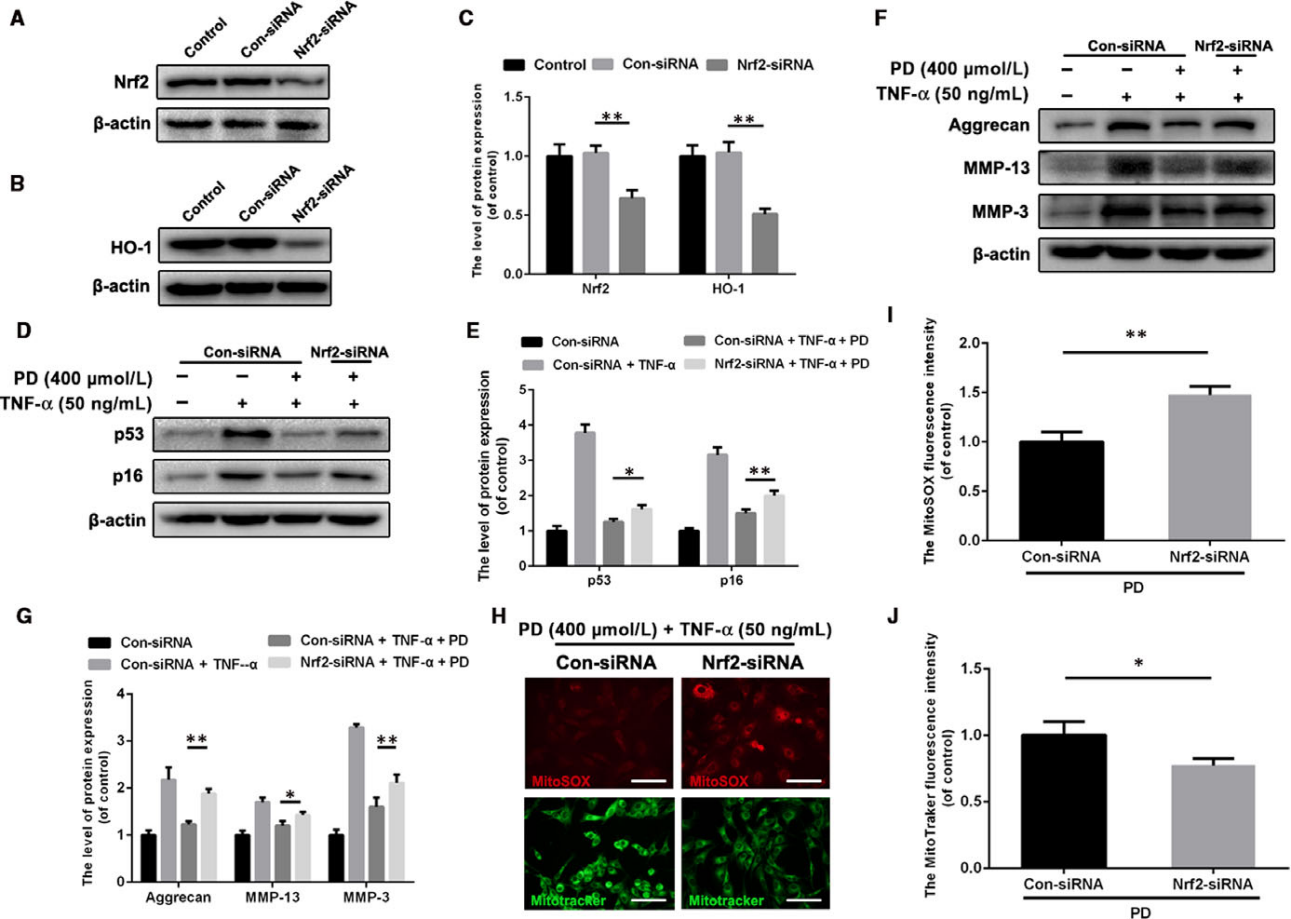
Knockdown of Nrf2 compromises the antisenescence, anti‐ECM disorder and antimitochondrial dysfunction properties of PD on NPCs. A‐C, Successful knockdown of Nrf2 suppressed the expression level of HO‐1. D, E, The expression of p53 and p16 in NPCs treated with or without pretreatment of PD for 2 h and another administration of TNF‐α for 72 h. F, G, The levels of ECM‐related proteins such as aggrecan, MMP‐13 and MMP‐3 were detected by Western blotting. H‐J, Representative images for MitoSOX and MitoTracker of PD and TNF‐α‐treated NPCs with or without Nrf2 knockdown. Scale bar: 50 μm. Data presented are the means ± SD of three independent experiments. *P < 0.05, **P < 0.01, n = 5

To research the role of Nrf2 in PD‐mediated protective effects on NPCs, we successfully established the Nrf2 knockdown NPCs model, with the lower level of HO‐1 (Figure 6A‐C). Then, we found that Nrf2 knockdown compromised the antisenescence property of PD in TNF‐α‐treated NPCs (Figure 6D,E). Also, Nrf2 was involved in the property of anticatabolism of PD on NPCs against TNF‐α, with more levels of MMP‐13 and MMP‐3 and the lower level of aggrecan (Figure 6F,G). Furthermore, we explored the role of Nrf2 in antimitochondrial dysfunction property of PD on NPCs. After Nrf2 knockdown, the Nrf2‐siRNA group showed stronger intensity of MitoSOX fluorescence and weaker intensity of MitoTracker fluorescence, indicating Nrf2 played a protective role in mitochondrial homeostasis (Figure 6H‐J).

### 3.7 PD ameliorates IVDD development and promotes ECM preservation while suppresses senescence in rat tail IVD‐punctured model in vivo

**Figure 7:**
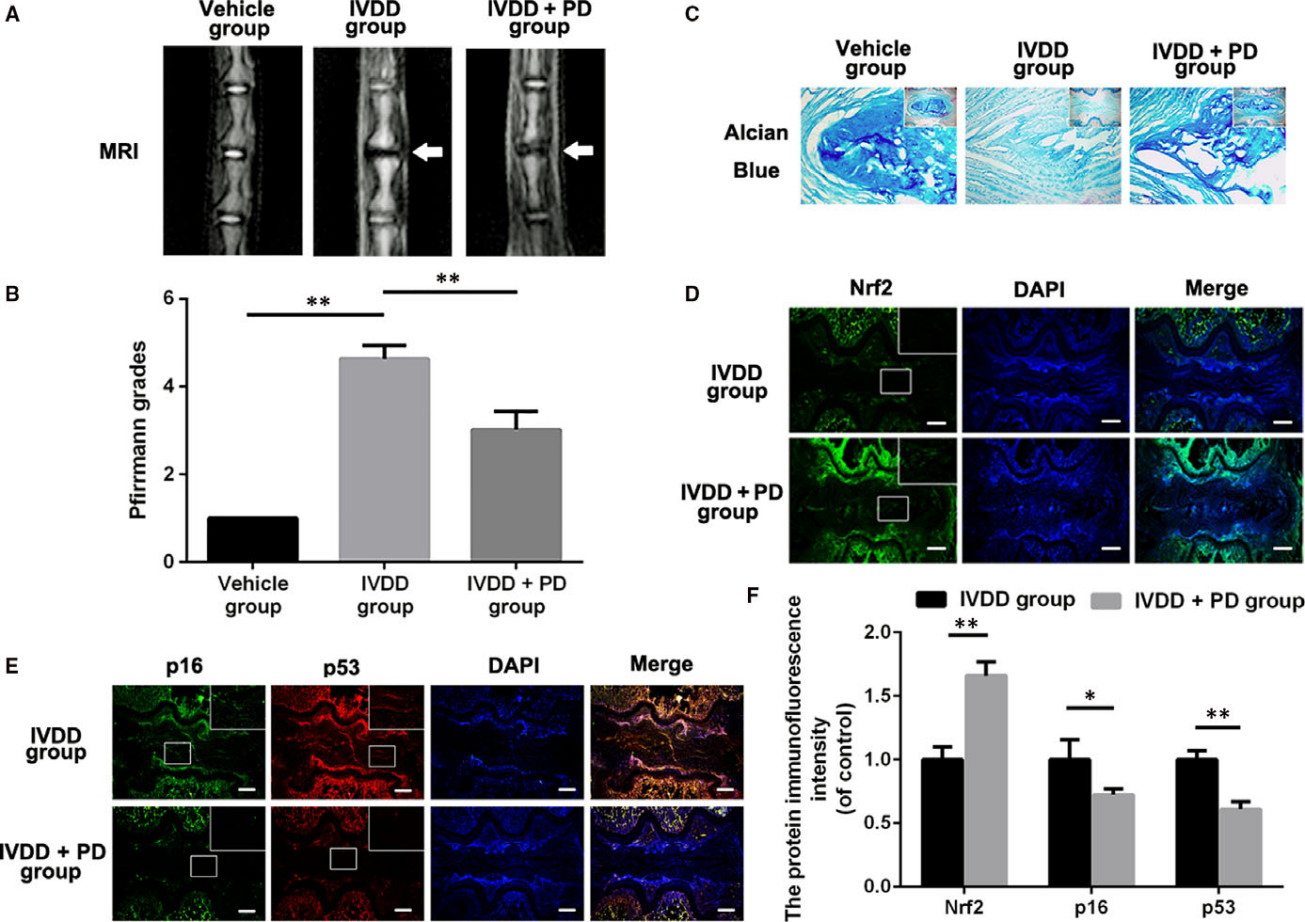
Polydatin (PD) ameliorates disc degeneration development in IVDD rat model in vivo. A, Magnetic resonance image (MRI) of IVDs in rats from different experimental groups. Loss of signal density was found in both IVDD and PD+IVDD group (white arrows). B, Diagrams showed the Pfirrmann grades of IVDs. C, Representative alcian staining of IVDs from different groups at 2 or 4 wk postsurgery. Scale bar: 100 μm. D, Representative images for Nrf2 in IVDs from the IVDD group and the PD+IVDD group. Scale bar: 100 μm. E, Representative images for p16 and p53 in IVDs from the IVDD group and the PD+IVDD group. Scale bar: 100 μm. F, The quantification of Nrf2, p16 and p53 fluorescence intensity of IVDs from the IVDD group and the PD group. Data presented are the means ± SD of three independent experiments. *P < 0.05, **P < 0.01, n = 5

Base on the results of vitro experiments, we explore the effects of PD on IVDD rat models. Intragastric administration of 50 mg/kg PD in 0.5% carboxymethylcellulose (CMC) or vehicle (0.5% CMC alone) once daily was performed as described above. Magnetic resonance imaging (MRI) obtained at 4 weeks after puncture showed that IVDD group exhibited severe loss of intensity in punctured IVDs but improved T2‐weighted signal intensities that could be observed in PD‐treated group (Figure 7A,B). Similarly, Pfirrmann grade scores, an evaluating method for IVDD, were significantly lower in the PD group than in the IVDD group at 4 weeks. Alcian blue staining, sensitive to proteoglycan and hyaluronic acid, is used to evaluate the degree of ECM content in rat NP and the morphology of the IVDs. As shown in Figure 6C, the blue colour of NP in the PD‐treated group was much stronger than the one in IVDD groups and there was more content of NP remained in the AF. Moreover, we investigated the expression of Nrf2 in NP to assess the effect of PD on apoptosis in NPCs. The images of immunofluorescence and corresponding quantification showed that PD could promote the level of Nrf2 in rat IVDs (Figure 6D,F). To verify that the administration of PD up‐regulated senescent markers in IVDs, we detected p53 and p16 using immunofluorescence in IVDs. Our results showed that the immunofluorescence intensity of p53 and p16 was elevated in PD group compared with vehicle group, suggesting senescence in IVDs was suppressed with the administration of PD (Figure 6E,F).

**Figure 8:**
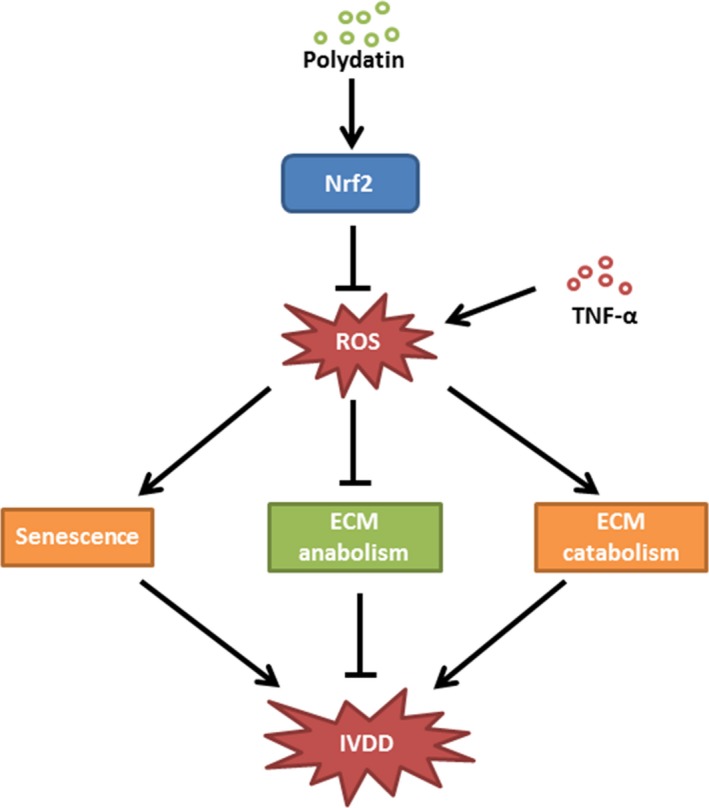
Schematics of mechanism of PD on IVDD. PD treatment suppresses Keap1 and enhances Nrf2 to activate Nrf2/HO‐1 signalling pathway to scavenge TNF‐α‐induced ROS, rescuing senescence and excessive ECM destruction in NPCs

## 4 DISCUSSION

Although the development of medicine is rapid now, the treatment for IVDD is still limited within relieving pain, neurotrophy and surgery in clinic.^27^, ^28^ Thus, it is urgent to explore new method in medical treatment to block the development of IVDD.

During IVDD, changes in intracellular physiological behaviours as well as ECM disorder in NPCs ultimately lead to biomechanically compromised IVDs. Mitochondria act as critical roles in metabolic regulation, redox balance and energy maintenance.^29^, ^30^ Previous studies have demonstrated that mitochondria are involved in apoptosis and ECM metabolism in NPCs,^11^, ^31^ indicating mitochondria may be the potential therapeutic target in ameliorating IVDD. Another marked feature of IVDD is that it is related with inflammatory cytokines such as TNF‐α. Strong evidence suggests that TNF‐α is highly expressed in degenerative NP and stimulates nerves causing low back pain.^32^ TNF‐α, produced by NPCs or other disc cells, contributes to an inflammatory microenvironment through NF‐κB and MAPK signalling pathways, disturbing ECM homeostasis and up‐regulating MMP and ADAMTS family members in NPCs.^33^, ^34^ In addition, TNF‐α promotes premature senescence of NPCs by attenuating the ability of ECM synthesis and up‐regulating levels of senescence markers p16 and p53.^6^ Also, TNF‐α causes mitochondrial dysfunction with intracellular ROS accumulation.^9^, ^35^ In short, TNF‐α may aggravate senescence and degradation of ECM via mitochondrial pathway in NPCs.

Polydatin, a natural polyphenol, has been reported to possess excellent anti‐aging property.^36^ Firstly, we confirmed the safe concentration of PD in NPCs. To reach the best effect of PD in vitro, we chose the most two highest concentration of 200 and 400 μmol/L as the experimental concentrations according to CCK8. In addition, we found that PD not only attenuated the premature senescence phenotypes but also rescued the disorder of matrix metabolism in NPCs treated with TNF‐α. Besides senescence, disorder of ECM metabolism in NPCs is also rescued by PD. In the current study, PD not only promoted the expressions of aggrecan and collagen II, two most prominent component of ECM, but also suppressed the ECM catabolic enzymes, MMP‐3, MMP‐13 and ADAMTS‐4, suggesting that PD maintains ECM metabolism homeostasis in NPCs under inflammatory microenvironment.

Senescence and cellular metabolism of ECM are implicated in mitochondrial dysfunction.^37^, ^38^ Exposure of cells to TNF‐α induces more generation of ROS, and overproduction of ROS can result in mitochondrial dysfunction.^39^, ^40^ Defective mitochondria with lower mitochondrial membrane potential can produce much reactive oxygen species (ROS), which promotes the production of intracellular malondialdehyde (MDA) that is the cell membrane lipid peroxidation product. In the current study, we found that PD alleviated the intracellular accumulation of ROS, improved the lowered mitochondrial membrane potential and rescued the impaired ATP production in NPCs treated with TNF‐α.

Although the beneficial roles of Nrf2 in anti‐inflammation and oxidative stress have been confirmed in previous studies, respectively, it is still uncertain whether PD activates Nrf2 in the rat NPCs at the safe concentrations. In our study, PD promoted the levels of nucleus Nrf2 in NPCs in a dose‐dependent manner, indicating PD could be considered as an effective Nrf2 agonist in treatment against IVDD. To explore the mechanism of PD on Nrf2 activation, we detected the expression of classical signalling pathway Nrf2/HO‐1 in NPCs treated with PD. Surprisingly, the level of Keap1 was down‐regulated by the administration of PD in NPCs, suggesting that PD leads to impaired Keap1 capacity of combining with Nrf2 and promotes the translocation of Nrf2 into nucleus. As we found, that is, motivation of HO‐1 induced by PD, the activation of Nrf2/HO‐1 in NPCs treated with PD can be confirmed.

To determine whether PD protected NPCs from senescence, ECM disorder and mitochondrial dysfunction via Nrf2/HO‐1 signalling pathway, we detected the expression of senescence, ECM disorder and mitochondrial condition in DHM‐treated NPCs with Nrf2 knockdown. In our study, Nrf2 knockdown abolished the protective effects of PD on senescence, with higher levels of p53 and p16 in Nrf2 knockdown group. Also, PD was able to rescue the TNF‐α‐induced ECM metabolism disorder as a Nrf2‐dependent manner in NPCs. As the Nrf2 mediated the protective effects of PD against senescence and ECM metabolism in TNF‐α‐treated NPCs, we presumed that it could also ameliorate mitochondrial dysfunction in TNF‐α‐treated NPCs. Correspondingly, we found that the administration of PD improves mitochondrial membrane potential and intracellular ROS via activating Nrf2 in NPCs under inflammatory and oxidative stress microenvironment induced by TNF‐α. Hence, our finding suggested that the protective effects of PD on NPCs could be mediated by mitochondrial homeostasis via Nrf2/HO‐1 signalling pathway.

As for the dose of PD in vivo, 50 and 200 mg/kg/d are the most commonly used doses in previous studies. In the present study, we found the oral dose of 50 mg/kg/d was enough to attenuate the degree of IVDD in rats.

The rat tail disc‐puncture model is widely applied for simulating the human IVDD condition.^11^, ^23^ The IVDD model exhibits loss of MRI signal density and less content of ECM in punctured IVDs. In our study, we found that PD not only rescued loss of MRI signal density but also protected against ECM degradation in degenerative IVDs. In addition, PD promoted the expression of Nrf2 in punctured IVDs, indicating PD might exert beneficial effects against IVDD via Nrf2 activation in vivo. As for the effects of PD on senescence phenotypes in rat IVDD models, we found that PD down‐regulated the level of p16 and p53 in IVDs punctured rats.

In summary, our findings suggest that PD prevents TNF‐α‐induced senescence and ECM disorder, by alleviating ROS‐mediated mitochondrial dysfunction via activating Nrf2/HO‐1 signalling pathway in rat NPCs. In addition, oral administration of PD on rats with IVDD not only rescues the loss of disc MRI signal density and ECM excessive destruction but also inhibits senescence in degenerative IVDs. These findings imply that PD may be a potential therapeutic agent in the treatment of IVDD.

## CONFLICT OF INTEREST

The authors declare no conflict of interests.
